# Psychosocial and organizational risk factors for doctor-certified sick leave: a prospective study of female health and social workers in Norway

**DOI:** 10.1186/1471-2458-14-1016

**Published:** 2014-09-29

**Authors:** Cecilie Aagestad, Reidar Tyssen, Håkon A Johannessen, Hans Magne Gravseth, Tore Tynes, Tom Sterud

**Affiliations:** Department of Occupational Health Surveillance, National Institute of Occupational Health, PO BOX 8149 Dep, NO-0033 Oslo, Norway; Department of Behavioral Sciences, Institute of Basic Medical Sciences, Faculty of Medicine, University of Oslo, Oslo, Norway

**Keywords:** Sick leave, Health and social workers, Psychosocial and organizational work environment, Violence

## Abstract

**Background:**

Doctor-certified sick leave differs substantially across sectors, and among health and social workers, in particular, there is an increased risk. Previous studies have shown that work environmental factors contribute to sick leave. Hence, the identification of specific organizational and psychosocial risk factors for long- term sick leave, taking into account potential confounding related to mechanical risk factors such as lifting and awkward body postures, will be of importance in the work of prevention.

**Methods:**

A randomly drawn population sample of Norwegian residents was interviewed about working conditions in 2009 (n = 12,255; response rate 60.9%). Female health and social care workers (n = 925) were followed in a national registry for subsequent sickness absence during 2010. The outcome of interest was doctor-certified sick leave of 21 days or more (long-term sick leave). Eleven work-related psychosocial and organizational factors were evaluated.

**Results:**

In total, 186 persons (20.1%) were classified with subsequent long-term sick leave. After thoroughly adjusting for competing explanatory variables, the most consistent predictors for long-term sick leave were violence and threats of violence (OR = 1.67; 95% CI 1.14–2.45). The estimated population attributable risk for violence and threats of violence was 13%.

**Conclusions:**

The present study among female health and social care workers revealed a substantial relationship between self-reported violence and threats of violence and subsequent long- term sick leave.

## Background

Doctor-certified sick leave differs substantially across sectors, and among health and social workers, in particular, there is an increased risk [1]. Long-term sick leave (LTSL) is considered a general indicator of ill health [2] and has been associated with a high risk of not returning to work [3, 4]. Previous studies have shown that work environmental factors contribute to sick leave [5, 6], and the identification of specific organizational and psychosocial risk factors for LTSL in the sector will be of importance in the work of prevention.

Several studies have reported that stressful factors at work predict sick leave [5–8]. In the general literature [5], as well as in the health and social sector, the main focus has been the study of factors related to the job demand–control (−support) model [9, 10]. A systematic review in 2004 reported that low job control was the best-documented psychosocial risk factor for increased sickness absence [5]. The effects of high demand, social support and the combination of high job demands and low control (job strain) were not conclusive, and few studies have addressed other work-related psychosocial factors [5]. A recent prospective study revealed that other factors, such as emotional demands and role conflict, may be important to study as potential risk factors for sick leave in the health and social sector [11]. However, these findings have yet to be confirmed in other prospective studies of the sector. Moreover, workers in the health and social sector have been found to be especially prone to adverse social behaviour such as violence and threats [12], and bullying has been reported as an important predictor of sick leave in prospective studies of health care workers [13, 14].

In addition to psychosocial stressors, organizational factors such as shift work [15], reorganization and other organizational changes are prevalent in the health sector [16]. In Norway, reorganizations due to hospital mergers have been prevalent in recent years; in the last 10 years, 90% of public hospitals were involved in one or more mergers [17]. Such organizational changes have been found to be positively related to distress [16] and have led to an increase in the risk of sick leave in the health-care sector [16, 18].

Several prospective studies of health and social care workers have examined the impact of psychosocial risk factors for doctor-certified LTSL [11, 14, 19]. However, these studies have not included organizational work environment factors, nor have they taken into account potential confounding related to mechanical risk factors such as lifting and awkward body postures. Mechanical risk factors are prevalent in the sector [20], and have been found to predict LTSL in previous prospective studies [21, 22]. To identify which factors contribute the most, we argue that, in addition to factors related to the traditional demand–control (−support) model [23], less frequently studied factors, such as emotional demands, role conflict, adverse social behaviour and organizational factors at work, should be included and studied in a large adjusted statistical predictor model of doctor-certified sick leave. In addition, by combining data on prevalence and risk, the present study enabled us to estimate the proportion of LTSL cases in the population attributable to psychosocial exposures at work; to our knowledge, only one previous study has provided such information [12].

Based on a nationwide cohort of female health and social care workers, the aim of this study was to prospectively analyse the impact of several work-related psychosocial and organizational risk factors for LTSL (doctor-certified sick leave ≥21 days), taking into account perceived mechanical exposures.

## Methods

Data were provided from the nationwide study of living conditions/work environment conducted by Statistics Norway. Data were collected during the period 22 June 2009 to 9 January 2010, by personal telephone interviews (0.5% of completed interviews were face-to-face). Prior to telephone contact, potential respondents were informed by mail about the study, the topic of the study and data/privacy protection. The survey was carried out by Statistics Norway according to statutory rules. Statistics Norway has appointed its own privacy ombudsman, approved by the Norwegian Data Inspectorate. All respondents gave their informed consent prior to their inclusion in the study.

### Population

The study population was women working in the health and social care sector (Table 1). The sample consisted of few men (14%), and we therefore decided to exclude men from the analyses. The study population was adopted from a population survey where eligible respondents were community-living Norwegian residents aged 18–69 years. In 2009, a gross sample of 20,136 was randomly drawn from this population. Of these, 7881 did not respond at baseline, and the most important reason was that the interviewer was unable to get in touch with the respondents despite several attempts (19%), 16% did not want to participate and 3% were prevented from participation. A total of 12,255 persons were interviewed (61%), and the baseline sample was compared with the gross sample according to the benchmarks of age, sex and region, and no major differences were detected [24].

**Table 1:** **Occupational groups and the associated risk of LTSL during follow-up**

Occupation	N	Distribution (%)	Cases n ^*^	Cases (%) (95% CI)
Total	925		186	20.1 (17.5–22.7)
Registered nurse	271	29.3	49	18.1 (13.8–23.2)
Physical therapist. Radiographer. Health worker with college education	63	6.8	11	17.5 (8.2–28.4)
Social worker. Social educator	99	10.7	25	25.3 (16.7–34.2)
Nursing and care assistants	409	44.2	82	20.0 (16.1–23.9)
Doctors’/dentists’ assistant. Pharmacy technician	83	9	19	22.9 (13.1–31.8)

*LTSL, long term sick leave ≥21 days during the 1- year follow–up.

Data on sick leave were obtained by a merger of the nationwide survey of living conditions/work environment and the national registry of social transfer payments. The registry includes all economically active individuals aged 16–69 in Norway (i.e., those with a minimum of 4 hours of paid employment per average working week) in the reference period. Female respondents who were in paid work for at least 1 hour during the reference week or temporarily absent from such work (both in 2009) and registered with a health and social care occupation constituted the follow-up sample in this study (n = 925). There was no significant difference in the prevalence of sick leave between the groups.

### Design

The study was designed as a 1-year follow-up of a population-based sample of female workers in the health and social care sector. Information on sickness absence (days lost) was registered for the year after collection of the questionnaire data, i.e. in 2010.

### Sick leave

In Norway, employees are entitled to use a personal declaration for sick leave of up to 3 days or a total of 8 days spread over four different occasions during a 12-month period, depending on their employer’s settlement with the Norwegian Labour and Welfare Organization. In addition, if the workers child is sick, the employee has the right to stay at home for 10–15 days, depending on the number of children. This number is doubled for single lone parents. If the employee is sick beyond the personal declaration days, or if the severity of the illness requires it, then doctor-certified sick leave is required. In Norway, the employee are entitled to sick-leave benefits equal to 100% of past earnings from day one, for the first year of absence. The first 16 days are paid entirely by the employer. For longer periods, the government pays the wages. Because minor health problems such as influenza and the ability to stay at home with sick children are covered by the personal declaration days, we believe that doctor-certified sick leave for 21 days or longer captures more serious sickness.

### Long-term sick leave

In this present study, LTSL was defined as medically confirmed sick leave for 21 or more actual working days during 2010, the year after the initial survey was undertaken.

### Predictors

Psychosocial factors were measured with six scales (quantitative demands, role conflict, supportive leadership, job control, emotional demands, possibilities for development) and two single items (bullying, violence and threats of violence). Cronbach’s alpha (α) was estimated for each scale. The scale *quantitative demands* at work was measured with two items from QPS Nordic (Nordic Questionnaire for Psychological and Social Factors at Work) [25] (α = 0.70): (i) “How often do you have to work quickly?”; and (ii) “How often do you have too much to do?” Answer categories were “very seldom or never”, “rather seldom”, “sometimes”, “rather often” and “very often or always”. *Role conflict* was measured with three items from QPS Nordic [25] (α = 0.64): (i) “How often do you receive contradictory requests from two or more people?”; (ii) “How often are you given tasks without being given sufficient tools and resources to complete them?”; and (iii) “How often do you have to do things that you think should be done in a different way?” Answer categories were “very seldom or never”, “rather seldom”, “sometimes”, “rather often” and “very often or always”. *Supportive leadership* was measured with three items from QPS Nordic [25] (α = 0.70): (i) “If needed, how often can you get support and help from your immediate superior with your work?”; (ii) “Does your immediate superior appreciate your achievements at work?”; and (iii) “Does your immediate superior treat employees fair and impartially?” Answer categories were “very seldom or never”, “rather seldom”, “sometimes”, “rather often” and “very often or always”. *Job control* was measured with two questions from QPS Nordic [25] and two questions developed by Statistics Norway [24] (α = 0.71): (i) “To what extent can you decide the pace at which you work?”; (ii) “To what extent can you influence decisions that are important to your work?”; (iii) “To what extent are you free to decide how to go about your work?”; and (iv) “To what extent are you free to decide your own tasks?” Answer categories were “to a very great extent”, “to a great extent”, “to some extent”, “not really” and “hardly at all”. *Emotional demands* was measured with two items developed by Statistics Norway [24] (α = 0.69). (i) “In your work, to what extent do you need to deal with strong feelings such as sorrow, anger, desperation, frustration, and so on from customers, clients, or other people who are not employed at your workplace?” Answer categories were “to a great extent”, “to some extent”, not really” and “not at all”. (ii) “In your work, to what extent do you need to conceal negative feelings such as anger, irritation, frustration, and so on for customers, clients, or other people who are not employed at your workplace?” Answer categories were “to a very great extent”, “to a great extent”, “to some extent”, not really” and “not at all”. For these work-related psychosocial factors, the mean scale score was converted into three categories: low (1.0–2.0), medium (2.1–3.0) and high (3.1–5.0). *Possibilities for development* was measured with two items developed by Statistics Norway [24] (α = 0.72): “In your job, how good are your opportunities to:” (i) “develop your skills in the areas that interest you?”; and (ii) “make use of the skills, knowledge, and experience that you have gained through your education and past work?” Answer categories were “very good”, “good”, “poor” and “very poor”. The mean of the scale score was converted into three categories: low (1.0–1.5), medium (1.6–2.0) and high (2.1–4.0). All variables were coded so that high scores assumed negative exposure, such as high quantitative demands, high role conflict, low job control, low supportive leadership, low emotional demands and low possibilities for development. *Bullying* was measured with two items developed by Statistics Norway [24]: “Do you yourself sometimes get bothered or teased in an unpleasant way by your colleagues?” and “Do you yourself sometimes get bothered or teased in an unpleasant way by superiors?” Answer categories were “yes, once or more a week”, “yes, once or more a month” and “no”. The items were recoded and collapsed into one dichotomous variable (yes = 1, no = 0). *Violence and threats of violence* was measured with three items developed by Statistics Norway: “Over the past 12 months have you been the victim of violence at the workplace that caused visible marks or physical damage?”, “Over the past 12 months have you been the victim of violence at the workplace that did not cause visible marks?” and “Over the last 12 months have you been threatened at the workplace in such a way that you felt scared?” Answer categories were “yes” and “no”. The items were computed into one dichotomous variable (yes at any item = 1, no = 0).

Organizational factors were measured with three items developed by Statistics Norway [24]. *Shift work*: “What are your normal working hours?” The answer categories were “daytime between 6 am and 6 pm”, “shift or rota work” and “other arrangement”. The last two categories were computed into “shift work”, and the variables were dichotomized into one variable (daytime = 0, shift work = 1). *Downsizing*: “Has the company where you currently work implemented downsizing at any point during the past three years?” *Reorganization*: “Has the company carried out any restructurings over the past three years that have affected your work situation, but have not involved staff cuts?” Answer categories in both items were “yes, in my department”, “yes, in other departments at the company” and “no”. The answer categories for downsizing and reorganization were each computed into a dichotomous variable (yes = 1, no = 0).

Potential confounders such as *age and educational level* were based on administrative registry data. *Occupation* was based on an open questionnaire and coded by Statistics Norway into a professional title, in accordance with the International Standard Classification of Occupations (ISCO-88). The variable *chronic health complaints* was measured with the question: “Do you have any long-term illnesses or health problems? This includes any illnesses or problems that are seasonal, or that are intermittent. The prerequisite is that the condition must have lasted, or be expected to last, at least 6 months.” *Disability* was measured with a single item: “Are you disabled, or do you suffer pain as a result of an injury? This includes pains that are intermittent.” *Smoking* was measured with two items: “Do you sometimes smoke?” “Yes” respondents were asked: “Do you smoke every day or occasionally?” These variables were recoded into regular smokers vs. non- or occasional smokers. *Perceived mechanical workload* (mean) was measured with seven items: neck flexion, hands above shoulders, hand/arm repetition, squatting/kneeling, standing, work with upper body bent forward, and awkward lifting. The answering categories were “almost all the time”, “3/4 of the time”, “half of the time”, “1/4 of the time” and “very little of the time”. The variables that have been shown to predict LTSL are described in greater detail elsewhere [22].

### Statistics

The associations between work environment and LTSL were calculated by logistic regression analyses as odds ratios (ORs) with 95% confidence intervals (CIs), with adjustment for potential confounders. Three models were evaluated. In model 1, we adjusted each variable for age and registered LTSL in 2009. In model 2, further adjustments were made for educational level, chronic health complaints, disabled/injured and smoking. In the fully adjusted model (model 3), further adjustments were made for perceived mechanical exposures. To limit the potential for overadjustment in model 2 and model 3, each work-related psychosocial predictor was only adjusted for other work-related predictors that were first estimated to exert an influence above a certain threshold level. This estimation was made *a priori*, based on the following procedure suggested by Rothman et al. [26] and applied to the baseline data. In the first step, a crude OR was estimated separately for each work-related factor. In the second step, each of the other work-related variables was entered one at a time. If the inclusion of a potential confounder resulted in a change in the OR of 10% or more, that variable was treated as a real confounder in the multiple regression models. In addition, we did separate analyses of respondents with no LTSL registered in 2009. Finally, we did separate analyses by excluding respondents who worked less than 100 working days in 2009 and 2010. All statistical analyses were conducted with PASW Statistics package (formerly SPSS), version 20.0 (IBM, Armonk, NY, USA).

For statistically significant work-related factors in the regression analyses in the three models, we calculated the population-attributable risk estimates (PAR) with a 95% CI. In contrast to OR estimates, the PAR estimate combines data on prevalence and a measure of association to provide a quantitative estimate of the proportion of cases in the population that are attributable to a particular exposure. The method is described in detail by Natarajan et al. [27].

## Results

During the follow-up period, 925 people participated in the study, and 186 (20.1%) were classified with LTSL. Tables 1 and 2 describe the distribution of socio-demographic and health variables at baseline and the associated risk of LTSL at follow-up. The risk was higher among workers in the 25–34-years age group, among persons with a chronic health complaint (27.2% vs. 18.3%), for those who were disabled/injured (28.7% vs. 18.9%), for regular smokers (27.5% vs. 18.3%) and for those having LTSL during the baseline year (46.3% vs. 13.3%). Table 3 shows the results of multiple logistic analyses, with baseline psychosocial and organizational risk factors as the predictors and LTSL during the follow-up period as the outcome. In model 1 (adjusting for sick leave during the baseline year and age), those reporting violence and threats of violence and those reporting bullying had a higher risk for LTSL. A non-significant but elevated OR was found for high role conflict and low supportive leadership. In the fully adjusted model 3, those reporting violence and threats of violence had a significantly higher risk for LTSL (OR = 1.67; 95% CI 1.14–2.45). Bullying was no longer a significant predictor when adjusted for violence and threats of violence, reorganization and supportive leadership. We also analysed violence and threats of violence separately. The fully adjusted OR for violence was 1.51 (95% CI 1.02–2.25), and the OR for threats of violence was 1.19 (95% CI 0.70–2.01). We tested for a dose–response association by entering the variables as continuous. None of the variables were significant.

**Table 2:** **Distribution of the socio-demographic variables and health variables at baseline and the associated risk of LTSL**

	N	Distribution (%)	Cases n*	Cases % (95% CI)	*p§*
**Total**	925		186	20.1 (17.5–22.7)	
**Age**					0.09
17–24 yr	73	7.9	8	11 (2.8–17.7)	
25–34 yr	170	18.4	44	25.9 (19.5–33.2)	
35–44 yr	251	27.1	50	20 (14.8–24.9)	
45–54 yr	229	24.8	48	20.9 (15.9–26.7)	
55–69 yr	202	21.8	36	17.8 (12.6–23.3)	
Missing	0				
**Education level**					0.029
Basic school level	83	9.0	8	9.6 (3.1-16.2)	
Upper secondary education. not finished	98	10.6	18	18.4 (10.6-26.2)	
Upper secondary education	307	33.2	77	25.1 (20.2-29.9)	
Universtity/college 4 y	406	43.9	78	19.2 (15.4-23.1)	
Universtity/college 4 y+	22	2.4	5	22.7 (3.7-42.7)	
Missing	9	1.0			
**Smoking**					0.005
No	725	78.4	133	18.3 (15.5–21.3)	
Yes	193	20.9	53	27.5 (20.7–33.4)	
Missing	7	0.8			
**Chronic health complaints**					0.013
No	647	69.9	112	17.3 (14.3–20.3)	
Yes	272	29.4	74	27.2 (21.8–32.6)	
Missing	6	0.7			
**Disabled/injured**					0.001
No	797	86.2	151	18.9 (16.2–21.7)	
Yes	122	13.2	35	28.7 (20.7–37.1)	
Missing	6	0.7			
**Long-term sick leave in 2009**					0.001
No	721	77.9	96	13.3 (10.9–15.9)	
Yes	188	20.3	87	46.3 (39.0–53.4)	
Missing	16	6.8	3		

*LTSL, long term sick leave ≥ 21 days during the 1- year follow–up, §Chi-square test.

**Table 3:** **Multiple logistic regression: LTSL during follow-up regressed on psychosocial and organizational risk factors measured at baseline**

	N	Distribution (%)	Cases n ^*^	Cases (%)	Model #1 OR (95% CI) †	*p*	Model #2 OR (95% CI) ‡	*p*	Model #3 OR (95% CI) ¥	*p*
**Quantitative demands**	921		186	20.2						
Low	90	9.8	13	14.4	1.00		1.00		1.00	
Medium	226	24.5	39	17.3	1.12 (0.55-2.29)	0.761	1.10 (0.53-2.29)	0.803	0.98 (0.51-1.90)	0.966
High	605	65.7	134	22.1	1.39 (0.72-2.65)	0.325	1.40 (0.71-2.73)	0.328	1.20 (0.60-2.02)	0.764
**Role conflict**	923		186	20.2						
Low	469	50.8	85	18.1	1.00		1.00		1.00	
Medium	330	35.8	66	20	1.04 (0.71-1.53)	0.839	0.98 (0.67-1.45)	0.930	0.95 (0.64-1.40)	0.786
High	123	13.3	35	28.5	1.61 (0.98-2.64)	0.059	1.43 (0.86-2.36)	0.169	1.27 (0.75-2.13)	0.374
**Emotional demands**	922		186	20.2						
Low	136	14.8	31	22.8	1.00		1.00		1.00	
Medium	304	33	52	17.1	0.69 (0.40-1.17)	0.743	0.73 (0.43-1.25)	0.253	0.71 (0.41-1.22)	0.215
High	482	53.3	103	21.4	0.84 (0.51-1.38)	0.953	0.87 (0.53-1.43)	0.574	0.83 (0.50-1.36)	0.453
**Job control**	918		186	20.3						
High	138	15	25	18.1	1.00		1.00		1.00	
Medium	452	49.2	89	19.7	1.09 (0.65-1.85)	0.738	1.03 (0.61-1.76)	0.907	0.99 (0.59-1.70)	0.989
Low	328	35.7	72	22	1.19 (0.69-2.05)	0.529	1.20 (0.69-2.08)	0.517	1.08 (0.62-1.89)	0.778
**Supportive leadership**	917		186	20.3						
High	575	62.7	104	18.1	1.00		1.00		1.00	
Medium	220	24	50	22.7	1.24 (0.82-1.87)	0.303	1.24 (0.82-1.88)	0.306	1.01 (0.69-1.49)	0.942
Low	122	13.3	32	26.2	1.55 (0.94-2.54)	0.800	1.54 (0.93-2.53)	0.091	1.27 (0.79-2.03)	0.320
**Possibilities for development**	923		186	20.2						
High	350	37.9	62	17.7	1.00		1.00		1.00	
Medium	382	41.4	82	21.5	1.15 (0.78-1.71)	0.474	1.17 (0.78-1.74)	0.442	1.13 (0.76-1.69)	0.558
Low	191	20.7	42	22	1.30 (0.81-2.07)	0.284	1.30 (0.80-2.09)	0.291	1.23 (0.76-1.99)	0.406
**Bullying**	916		186	20.3						
No	891	97.2	176	19.8	1.00		1.00a		1.00a	
Yes	25	2.7	10	40	3.10 (1.30-7.4)	0.011	2.44 (0.94-6.33)	0.066	2.23 (0.85-5.85)	0.104
**Violence and threats for violence**	919		186	20.2						
No	692	75.3	125	18.1	1.00		1.00		1.00	
Yes	227	24.7	61	26.9	1.75 (1.20-2.55)	0.004	1.69 (1.15-2.47)	0.007	1.67 (1.14-2.45)	0.009
**Shift work**	924		186	20.1						
No	350	37.8	65	18.6	1.00		1.00		1.00	
Yes	574	62.1	121	21.1	1.18 (0.82-1.69)	0.373	1.16 (0.80-1.67)	0.437	1.05 (0.72-1.53)	0.482
**Downsizing**	899		183	20.4						
No	614	68.3	113	18.4	1.00		1.00		1.00	
Yes	285	31.7	70	24.6	1.28 (0.89-1.84)	0.179	1.19 (0.82-1.73)	0.359	1.15 (0.79-1.67)	0.463
**Reorganization**	903		186	20.6						
No	581	63.3	107	18.4	1.00		1.00b		1.00b	
Yes	322	35.7	79	24.6	1.27 (0.90-1.81)	0.179	1.12 (0.77-1.65)	0.551	1.11 (0.75-1.62)	0.613

Note. 1 reference value = not exposed or exposed very little of the work-day; †adj. for age, and long term sickleave in 2009, ‡ further adj, for education, chronic health complaints, disabled-/injured, smoking and for work related psychosocial exposures yielding a 10% change of OR) a, adj, for violence and threats of violence, reorganization, supportive leadership, b, adj. for downsizing, ¥ further adj. for mechanical exposures. ^*^LTSL, long term sick leave ≥21 days during the 1- year follow up.

When excluding respondents with LTSL in 2009 from the analyses, minor changes in the estimates were detected. In addition, when we validated the findings by excluding respondents who worked less than 100 working days in 2009 and 2010, minor changes in the estimates were detected. The population risk of LTSL attributable to the work-related psychosocial factors is shown in Table 4. Based on the fully adjusted model, the population risk of LTSL attributable to violence and threats of violence was 13%.

**Table 4:** **Calculated population attributable risk (PAR %) for LTSL based on the statistically significant ORs from model #1, model# 2 and model #3 in Table** 3

	Model #1†	Model #2‡	Model #3¥
**Risk factors**	PAR (95% CI)	PAR (95% CI)	PAR (95% CI)
Violence and threats of violence	14 (2.9-25.4)	13.4 (2.1-25.0)	13.1 (1.8-24.9)
Bullying	3.6 (0.2-8.0)		

†adj. for age, and long term sick leave in 2009, ‡ further adj, for education, chronic health complaints, disabled-/injured, smoking, and for work related psychosocial exposures yielding a 10% change of OR, ¥ further adjusted for mechanical factors. LTSL, long term sick leave ≥ 21 days.

## Discussion

In this 1-year prospective study of health and social workers in Norway, we found that exposure to violence increased the risk for LTSL, and we estimated that about 13% of the cases with LTSL in 2010 were attributable to violence and threats of violence at the workplace. We found that the risk for LTSL associated with psychosocial and organizational factors was not influenced by adjustment for mechanical risk factors.

Threats of violence, and in particular, violence, stand out as important risk factors for LTSL in the present study, and our results are in line with a study of human service workers in Denmark [12]. In the Danish study, it was estimated that elimination of exposure to violence and threats would have potentially reduced sickness absence days by 10% [12], which is fairly consistent with our finding of 13%. It is well known that physical violence has a direct consequence for worker injuries [28], and it has been associated with mental health problems [29] and musculoskeletal pain [30]. Musculoskeletal and mental health disorders account for a substantial part of doctor-certified sickness absence in Norway, and often lead to LTSL [31, 32]. To the best of our knowledge, this is the first prospective study to examine the impact of violence on doctor-certified sick leave.

Bullying was a significant predictor in model 1. Bullying has been reported to predict sick leave in two earlier prospective studies among health care workers [13, 14]. However, when we adjusted for supportive leadership, violence and threats of violence and reorganization the association was no longer significant. Hence, the non-significant result of bullying in model 2 may be due to overadjustment or low statistical power.

We found no significant association between role conflict and supportive leadership and LTSL, which is in contrast to our recent prospective study of the general working population, where those reporting high role conflict and low supportive leadership showed a significant excess risk for LTSL [33]. However, in this present study, both role conflict and supportive leadership had non-significant but elevated risk estimates in model 1. This could imply a type 2 error due to low statistical power. The finding of no associations between emotional demands and LTSL is in contrast to the findings of Clausen and co-workers [11]. In addition, those reporting high emotional demands were found to be at an excess risk for LTSL in our recent population study [33]. However, we did not find an effect among women [33], which is in line with the results of this present study.

We investigated the importance of the core dimensions of the job demand–control model as risk factors for LTSL. In line with two prospective studies among health and social workers from Denmark [11, 12], no significant associations between high quantitative demands and LTSL were found. Job control did not predict LTSL in this present study, which is in contrast to the Danish findings [11, 12]. However, our result is in line with a prospective study among Norwegian nurse aides [34]. Possibilities for development was not a significant predictor, which is in line with the findings of a recent Danish study [12].

We investigated the importance of a range of potentially important organizational factors such as shift work, reorganization and downsizing. No significant association between any of these factors and sick leave was found. In a recent systematic literature review, inconclusive evidence for an association between shift work and sickness absence was reported [15].

A strength of this study was the use of a large nationwide survey using random sampling prospectively linked to registered sickness absence data, with practically no loss to follow-up. LTSL, as the outcome variable, was registered during the year after work-related psychosocial and organizational exposures were measured by a survey questionnaire. The use of different sources of measures excludes the potential for common method bias [35]. Additional analyses among respondents without LTSL in 2009 yielded minor changes in the estimates compared with the full study sample, which indicates that reversed causality is not a likely explanation for the observed associations. The study had a fairly high response rate. The response rate was 61%. However, when evaluating potential systematic differences between responders and non-responders, Statistics Norway found no differences across the benchmarks of age, sex and region [24]. On the other hand, we do not know whether people with poor health, or elevated risk for sick leave, were less likely to respond at baseline, which may have led to biased and attenuated estimates and thus threatened the internal validity. However, studies have shown that some differences in participation in questionnaire surveys related to socio-demographic variables and health status do not produce biased risk estimates [36]. The ideal time-lag for longitudinal job stress research, has remained a long standing methodological issue, and definitive insights remain indefinable to-date [37]. The follow up time in this study was 1-year. One could argue that a longer follow up time would be more appropriate, because of more sufficient time of exposure to create effects on the outcome variable. However, a longer follow-up time could be considered a limitation as well, due to the fact that during a longer time period between exposures and effect, the levels of exposure might have changed for some participants, which may lead to an underestimation of the effect sizes [12].

Because of data protection issues, we could not obtain information regarding diagnosis for the medically confirmed sick leave. Moreover, we could not obtain data on the number of sick-leave periods, the length of each period or start and stop dates for a given period. The cut-off chosen to define LTSL (≥21 days) during a calendar year was considered a reasonable proxy for LTSL and one that allowed us to compare our findings with those of other studies of psychosocial predictors of LTSL. In addition, it can be considered a limitation of this study that we could not analyse the associations between the psychosocial work environment exposures and risk of LTSL separately for each of the five occupational groups (listed in Table 1) because of a lack of statistical power in the dataset.

## Conclusions

The present study revealed a substantial relationship between self-reported violence and threats of violence and subsequent LTSL among women in the health and social care sector. Interventions aimed at reducing LTSL in the sector may benefit from focusing on protection against violence and threats of violence in the workplace.

## Abbreviations

LTSL: Long- term sick leave
QPS Nordic: Nordic questionnaire for psychological and social factors at work.
